# What Research Is Needed to Stop TB? Introducing the *TB Research Movement*

**DOI:** 10.1371/journal.pmed.1001135

**Published:** 2011-11-29

**Authors:** Christian Lienhardt, Marcos Espinal, Madhukar Pai, Dermot Maher, Mario C. Raviglione

**Affiliations:** 1 Stop TB Partnership, World Health Organization, Geneva, Switzerland; 2 McGill University, Montreal, Canada; 3 London School of Hygiene and Tropical Medicine, London, United Kingdom

## Abstract

Christian Lienhardt and colleagues describe the development of the TB Research Movement, which aims to create a framework for concrete actions to harmonize and synergize TB research efforts globally.

**Summary Points**

- Current tuberculosis (TB) control tools are insufficient to confront the global burden of TB. Novel tools and interventions are highly needed.
- The Stop TB Partnership and the WHO Stop TB Department have launched the *TB Research Movement*, with the aim of boosting TB research and accelerating progress in TB control towards international targets.
- In this paper, we describe the development of the Research Movement strategic plan, highlighting progress in its two key components: (1) the analysis of the global funding landscape for TB research, and (2) the development of a global TB research agenda.
- Through this strategic plan, the TB research movement is creating a framework for concrete actions to harmonize and synergize TB research efforts globally, so that the poor and vulnerable populations burdened by TB will reap the dividend of less TB through more research and innovation.

## Introduction

With 9.4 million new cases and 1.7 million deaths worldwide in 2009, tuberculosis (TB) constitutes an unacceptable burden of human suffering and loss [1]. The tools available for TB control are old, lack effectiveness, and are not readily accessible in many settings: the diagnosis of pulmonary TB still relies in most high-burden countries on sputum microscopy, a century old technology; treatment of tuberculosis is based on drugs that are over 40 years old and requires direct supervision to ensure full treatment adherence and prevent drug resistance; and the only TB vaccine (BCG), first used in 1922, has a variable protective efficacy in adults. Novel tools are needed for better TB care and control worldwide [2].

Research has a key role to play in meeting health and development goals. Based on the World Health Organization's (WHO) Stop TB Strategy, the Stop TB Partnership has developed the Global Plan to Stop TB 2011–2015, which lays out the activities to be achieved by 2015 towards elimination of TB (defined as ≤1 TB case per million population per year) by 2050 [3]. The plan sets out a roadmap for halving TB prevalence and deaths globally by 2015, compared with 1990 levels. However, while it is estimated that the incidence rate of TB has been falling globally since 2004, the present rate of decline (less than 1% per year) is insufficient to reach the elimination goal by 2050 [1]. Any realistic prospect of achieving this goal depends both on the better and wider use of existing technologies and the development of revolutionary new technologies for TB control. This would be possible only through an acceleration of research across the continuum, from basic to implementation [4].

Recognizing this, the Stop TB Partnership and the WHO Stop TB Department have launched the *TB Research Movement*, with the aim of boosting TB research and accelerating progress in TB control towards international targets [5],[6]. We describe here the strategy developed to address the objectives (Box 1) of the Research Movement and the progress made over the last 2 years.

**Box 1. The TB Research Movement Objectives**

*Objectives of the Research Movement:*

1. To provide leadership and advocacy to mobilize increased resources in support of a coherent and comprehensive global TB research agenda to meet the Stop TB goals and targets; and
2. To provide a forum for funders and implementers of TB research to coordinate plans and actions, with the result of ensuring that research needs are addressed, opportunities identified, and gaps filled.

## The Research Movement Strategic Plan

The TB Research Movement is based at the Stop TB Partnership secretariat, housed by the WHO in Geneva, and works in close collaboration with the WHO Stop TB Department and with the Working Groups of the Stop TB Partnership. It operates as an umbrella for research-related issues at the Partnership, and receives advice from the Partnership Coordinating Board and the WHO.

The strategy plan developed to address the main objectives of the Research Movement has two major components: (1) the analysis of *global TB research funding* (aimed at estimating the funding gap); and (2) the development of a *global TB research roadmap* (representing a consensus on global needs across the TB research spectrum). To this end, the Research Movement has mobilized a broad alliance of stakeholders involved in TB research and development, including scientists involved in basic, applied, and operational research, TB control managers and public health officers, donors and aid agencies, and patients and community representatives.

### Analysis of Global TB Research Funding

**Figure 1:**
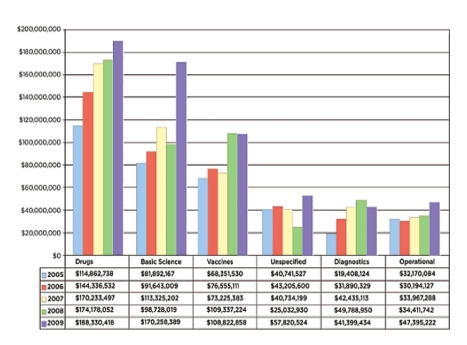
Investment in TB R&D by research category: 2005–2009 (from TAG report 2010). Reproduced with permission.

Mapping the research funding environment involves answering the questions “*who is funding TB research and development (R&D)?*”, “*what is being funded?*”, and “*how much is being granted?*”. For this, the Research Movement has joined with the Treatment Action Group's (TAG) efforts in evaluating the global landscape of funding in TB R&D, through a worldwide survey of funders and donors. This survey has been carried out regularly since 2005 and brings elements of responses to the questions above and helps monitor the trends in funding TB R&D internationally [7] (Figure 1).

The revised Global Plan to Stop TB 2011–2015 estimates that at least US$9.8 billion are needed in TB R&D over the next 5 years to reach the targets of 50% reduction in TB prevalence and mortality by 2015, more than twice those estimated in the initial Global Plan to Stop TB 2006–2015 (Table 1) [8]. Importantly, this updated Global Plan includes target investments for fundamental and operationalresearch, on top of the R&D for new drugs, diagnostics, and vaccines.

**Table 1:** Global Plan to Stop TB 2011–2015: total needs (US$ billion).

Plan Component	Total FundingRequired(% Total)
**Implementation**	**36.9 (79%)**
- DOTS	22.6 (48%)
- Drug-resistant TB	7.1 (15%)
- TB/HIV	2.8 (6%)
- Laboratory strengthening	4.0 (8%)
- Technical assistance	0.4 (1%)
**Research and Development**	**9.8 (21%)**
- Fundamental research	2.1 (5%)
- New diagnostics	1.7 (4%)
- New drugs	3.7 (8%)
- New vaccines	1.9 (4%)
- Operational research	0.4 (1%)
**All components**	**46.7 (100%)**

Adapted from reference [3].

**Figure 2:**
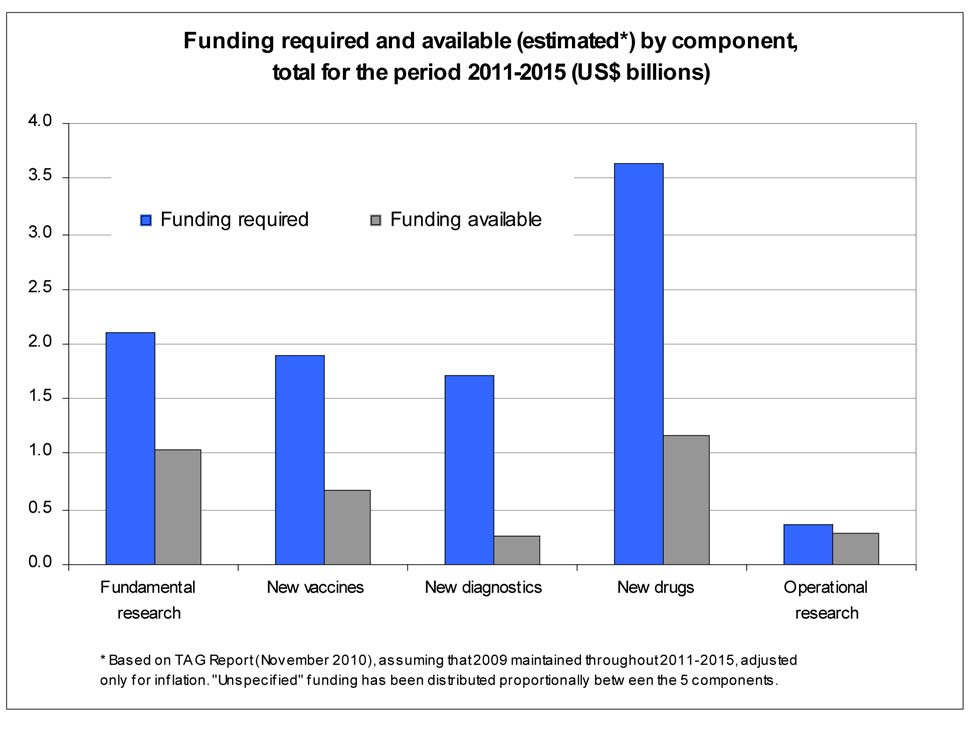
Funding required and available by research component 2011–2015.

From the above, the *research funding gap* can be estimated by comparing the results of the R&D funding survey to the research needs outlined in the Global Plan to Stop TB 2011–2015. Based on TAG Report 2010 [7], assuming that 2009 funding estimates are maintained throughout 2011–2015, and adjusting for inflation, the total funding gap for the next 5 years (2011–2015) is estimated at US$6.4 billion (64%) (Figure 2). The biggest gap in absolute terms is in R&D of new drugs, but the largest in relative terms (as a percentage of total funding required for that component) is R&D of new diagnostics. Remarkably, despite a significant boost in funding R&D for new tools in the past few years, TB research globally remains grossly underfunded, with a funding gap that is disproportionately greater for TB research (60%) than for implementation (35%) (Table 2).

**Table 2:** Funding required and funding available under two possible scenarios and likely funding gaps (US$ billions).

	*Scenario 1.*Funding maintainedat 2009/2010 levels^a^	*Scenario 2.*As for Scenario 1, but domestic funding for implementation increasesat rate of per capitaincome growth^b^
**IMPLEMENTATION**		
A. Funding required	37	37
B. Domestic (endemic country) funding available	21	23
- Brazil, China, India, South Africa and Europe	15	17
- Rest of world	5	6
C. External (donor) funding available if 2010 levels maintained^a^	3	3
D. Funding gap (A–B–C)	13	11
**RESEARCH AND DEVELOPMENT**		
E. Funding required	10	10
F. Funding available if 2009 levels maintained^c^	3	3
G. Funding gap (E–F)	6	6
**TOTAL FUNDING GAP**	19	17

a Adjusted only for inflation.

b GDP per capita in international dollars (purchasing power parity), forecast for the period 2011–2015 by the International Monetary Fund.

c Based on the TAG report (November 2010), assuming that 2009 levels are maintained throughout 2011–2015, adjusted only for inflation.

Adapted from reference [3].

### The Global TB Research Roadmap

In compiling a global TB research agenda, the fundamental question is “*what research is required to stop TB*?” This involves answering the questions of “*what are we researching in TB?*” and “*are we trying to answer the right questions?*”, i.e., “*are we doing studies in areas where evidence is lacking?*”. In addressing these questions, we needed to identify critical gaps in research that represent bottlenecks for development of new tools. This allowed the development of a coherent and comprehensive *global TB research roadmap* towards TB elimination that encompasses all aspects of research, from basic science for discovery, to development of new tools, and their optimal uptake for better TB control.

The steps in developing the global TB research roadmap include a series of consecutive activities that are described below: (1) an inventory of the research agendas; (2) the development of key research questions; and (3) the prioritization of research questions.

#### Inventory of TB research agendas

Over the past decade, a variety of research agendas has been developed by various groups. A systematic review of these TB research agendas was carried out to evaluate the main research questions and themes, assess the methods used to select priorities, and identify any consistent message emerging from these agendas [9].

The review identified 33 papers. The priority areas for research were: drug development (28 articles), diagnosis (27), epidemiology (20), health services research (16), basic research (13), and vaccine development (13) (Table 3). Research questions were usually quite broad in scope. The most focused questions were on treatment and prevention of multidrug-resistant TB and TB/HIV co-infection, reflecting the inefficiencies of sputum-smear microscopy and the limits of the currently recommended short-course chemotherapy, which is inefficient against drug-resistant forms of the disease and is difficult to combine with standard antiretroviral therapy. The importance of epidemiology and health system research probably reflects the need for studies to optimize the availability and cost-effectiveness of interventions for TB control. The relatively low priority assigned to basic research may indicate the difficulty of establishing an agenda in a field that is mostly investigator driven.

**Table 3:** Number of studies identifying priority topics for TB research in a systematic review of 33 articles with TB research priorities.

Research Topic	*n*
**Drug development and use (7 or more articles)**	**28**
Chemoprophylaxis effectiveness studies	9
Optimal length of drug treatment—new and old regimes	9
Development of new anti-TB drugs	7
Pharmacokinetics of standard drugs	7
Drug interaction studies (with concomitant antiretroviral use)	7
Pharmakokinetics of second-line drugs	7
**Diagnosis and diagnostic tests (8 or more articles)**	**27**
New diagnostic tests for active TB	14
New drug sensitivity testing methods	11
Evaluation of diagnostic pathway for the diagnosis of active TB	8
Biomarkers of successful treatment (for clinical or future trial use)	8
**Epidemiology and public health (5 or more articles)**	**20**
Accurate measurement of the global burden of TB disease	8
Determination of the role of social factors within communities on the risk of infection/transmission	5
Effect of treatment literacy programs on adherence and burden of disease	5
**Health services research (4 or more articles)**	**16**
Investigation of the causes of diagnostic delay	4
Modeling TB- associated costs/health service requirements	4
Role of patient groups in case finding	4
Best model for integrating TB and HIV services	4
Training requirements for staff providing TB care	4
**Basic science research (3 or more articles)**	**13**
Identification of host correlates of protection against TB disease	4
Understanding latent infection and latency	4
Understanding genetic and phenotypic markers of TB resistance	4
Development of an animal model that predicts treatment duration	4
**Vaccine development and use (2 or more articles)**	**13**
Development and trials of new TB vaccine	8

Source: Rylance et al. [9].

The methods used to identify priorities in these various agendas varied greatly. Most of these relied on expert meetings with consensus seeking, but few used objectively measurable criteria to select research priorities. Increased recourse to systematic reviews and use of clearly described and reproducible criteria to assess the importance of the research questions would greatly help in the establishment of research priorities.

#### Workshops to identify key research questions

Four workshops were organized in 2009 and 2010 to map out the landscape of TB research and identify gaps and priorities across the research continuum:

- Two workshops were organized on new diagnostics, drugs, and vaccines. These assembled scientists, program managers, public–private partnerships, representatives from civil society, donors, and members of the Stop TB Partnership Working Groups. The objectives were to review the progress achieved since 2006 in the Global Plan to Stop TB 2006–2015, and update the research activities needed for the development of new diagnostics, drugs, and vaccines to meet the targets of the Global Plan by 2015.
- A workshop on basic research for TB was organized in Bethesda, Maryland, United States, in March 2010, with the support of the National Institutes of Health/National Institute of Allergy and Infectious Diseases (NIH/NIAID) and TAG. The objective was to define the critical priority questions that need to be addressed in the *fundamental research area* to underpin the development of new drugs, diagnostics and vaccines. This workshop was a major step in reiterating the importance of fundamental science as the driver of innovation for improved TB control [10].
- A workshop was organized in May 2010 with the support of the Global Fund to Fight AIDS, Tuberculosis and Malaria to identify the operational (i.e., implementation, programmatic) research priorities to improve TB care and control. Five areas were identified: (i) access to, screening for, and diagnosis of drug-susceptible and drug-resistant TB; (ii) development of sustainable collaboration with all practitioners for TB care and control; (iii) prevention and treatment of TB in HIV-infected TB patients; (iv) optimal access to and delivery of treatment for drug-susceptible and drug-resistant TB; and (v) capacity building. Participants developed a list of operational research questions that were subsequently circulated to the Working Groups of the Stop TB Partnership for comments and suggestions [11]. One of the main outcomes of the workshop was the development of a document that lists the research priorities in the five key areas and provides, for each of these, a synopsis of the relevant methods and designs to address these research questions [12].

#### The prioritization of research questions

More than 250 research questions were identified within these workshops in the areas of fundamental science, R&D of new diagnostics, drugs, and vaccines, and operational and public health research. We set up a prioritization process to rank these questions using clearly defined and objectively measurable indicators, adapted from the Child Health Nutrition Research Initiative [13]. Prioritization was primarily based on the *value* that a scientific question is adding to the research area, how critical it is for the development of new tools and how it provides guidance for the implementation of these tools, to ultimately reduce morbidity and mortality due to TB. The priority ranking process was carried out independently by a group of 50 multi-disciplinary stakeholders representing a wide scope of research areas, public health, clinical care, and program management aspects.

#### Development of a roadmap for international TB research towards elimination

A coherent list of key research questions to be addressed for better TB control was established. We then requested experts to estimate the timeline under which these key research questions will be addressed in a chronological sequence, and their feasibility, so as to assess how the responses to these questions will fill the knowledge gaps and be conducive to further questions. The roadmap for international TB research is thus a living document that will be updated according to progress made. It is expected that the roadmap will provide a common framework for scientific disciplines to work concurrently and collaboratively towards better TB control, and will serve to promote TB research worldwide, including in high-burden countries.

The roadmap was presented and discussed at an international strategic meeting held in March 2011 in Bellagio, Italy, with the co-sponsorship of the Rockefeller Foundation. This meeting assembled major stakeholders, including key scientific thought leaders, representatives from nongovernmental organizations (NGOs), the vice ministers of health of Brazil and South Africa, and the top worldwide investors in TB research. The aim was to discuss the roadmap's vision of harmonized, synergistic, global TB research efforts through coordination of actions and funding. A summary of the main conclusions is provided in Box 2. In short, the Bellagio participants agreed that the Research Roadmap was a key vehicle to speed-up and coordinate TB research worldwide and that it be formally endorsed by the Coordinating Board of the Stop TB Partnership. It is expected that the roadmap will serve as a reference for activities carried out in the described research areas in support of the research objectives described in the Global Plan 2011–2015 and beyond.

**Box 2. Conclusions of the Bellagio Meeting (16 March 2011)**

The participants at the Bellagio meeting encouraged the Stop TB Partnership to endorse the Roadmap for International Research to Eliminate TB, publish it promptly as an independent document, and facilitate the execution of the following Action Plan:

1. Elaborate key areas of emphasis from the research roadmap to define an action plan for global TB research (including research advocacy);
2. Initiate consultations with countries, especially BRICS countries, researchers, policy makers, the private sector, and civil society to explore the key areas of emphasis/action plan and build ownership;
3. Match existing funded research with areas of emphasis to avoid unnecessary duplication, leverage existing resources and infrastructure to catalyse more effective collaborations;
4. Funders will establish a harmonization and coordination mechanism for research support.

## Discussion

Devising a creative research response to the global TB epidemic is a pressing health research issue. In the context of health and human development, research to accelerate progress in TB control will have a direct impact not only on decreasing suffering and saving lives, but also on alleviating poverty and promoting social and economic development.

Much progress has been made over the last decade in the development of new tools for better TB control after decades of neglect. The TB diagnostics pipeline has rapidly expanded, and a major breakthrough was the recent introduction of Xpert MTB/RIF, a molecular assay capable of diagnosing TB and the presence of rifampicin resistance in 100 minutes [14]. The pipeline of new TB drugs has substantially expanded with more than 15 compounds in preclinical and clinical development, including nine novel candidates currently in phase I and II trials [15]. Ten vaccine candidates have entered clinical trials, four of which are presently in phase II trials [16]. Through the commitment of public–private partnerships and the engagement of major public and private research donors, there is a potential for efficient new tools to be available before 2015. This is, however, insufficient. As suggested by a recent mathematical model, the possibility to effectively control and eliminate TB by 2050 would rely on the *combined and synergistic implementation of several novel strategies*, including improved diagnosis of drug-susceptible and drug-resistant TB, shorter treatment of overt TB cases (≤2 months), scaled-up treatment of latently infected persons (especially in high-risk populations), and mass vaccinations campaigns using a more effective vaccine [17]. This will happen only through synergistic efforts in all areas of R&D.

This formidable challenge will not be met without increased investment in fundamental research for a better understanding of the natural history of TB in humans [3]. Further progress is needed to develop a point of care diagnostic tool that would diagnose all forms of TB in all settings, including latent TB infection [18]. While novel drugs are reaching the late development phase, we still need to identify suitable combinations to treat optimally all forms of TB in all populations and risk groups, as well as latent TB infection [19]. Lastly, the search for highly effective vaccines for the prevention of TB in all populations needs to be continued and amplified [16]. For each of these tools, the development pathway has a high attrition rate, and the chances of a successful product emerging from the end of the development pipeline depend in large part on the number of potential products entering the pipeline [19],[20].

Research is also needed *downstream* to identify means to improve TB control with existing tools, and guide the uptake and scale-up of innovations within reinforced health systems in endemic countries [21]. In this, the WHO plays a crucial normative role in assessing the evidence to endorse (or not) the new tool(s) or intervention(s) and providing guidance on their implementation through policy recommendations and technical support. This process has been conducted recently for new molecular diagnostics [20], and needs to be pursued for the new anti-TB drugs that will soon become available [19]. For this to happen smoothly, global coordination of efforts is essential to ensure rapid transfer of products and innovations to endemic countries.

There are, however, major challenges to be overcome. In view of the current global economic situation and the likelihood that available support by current major donors may stagnate or even decline in the near future, there is a need to increase the number and diversity of donors, and make the most rational use of any dollar spent on research. Countries with a high TB burden, especially Brazil, Russia, India, China, and South Africa (BRICS), could play a major role in TB R&D through increased contribution, as exemplified by the recent declaration from the first BRICS Health Ministers Summit in Beijing, China, in July 2011 [22],[23], which recognized the “need to establish priorities in research and development” and called for “increased innovation” in TB, and for support of “transfer of technologies and innovation in a sustainable way”.

Further discussions are needed so that key donors in TB research develop a consensus on funding a harmonized global TB research agenda for the years to come, and to enable the much needed acceleration in the development of new tools for control and their rapid uptake in policy and practice. This can take the form of coordinated cross-disciplinary projects to expedite research in specifically identified key strategic areas that will lead to concrete public health outcomes. It is expected that the International Roadmap for Tuberculosis Research will serve as a framework for concrete actions to synergize TB research efforts globally and catalyze the development of new research collaborations to address difficult and yet unanswered questions in TB. The Research Movement can play a crucial role in leveraging existing resources and infrastructure to accelerate research for much needed progress in TB control towards achievement of international targets [2],[3].

## Abbreviations

R&D: research and development
TAG: Treatment Action Group
TB: tuberculosis
WHO: World Health Organization
